# Effectiveness of a New Self-Marking Technique in *Aedes aegypti* under Laboratory Conditions

**DOI:** 10.3390/insects13040379

**Published:** 2022-04-12

**Authors:** Gorgui Diouf, Momar Talla Seck, Assane Guèye Fall, Mireille Djimangali Bassène, Biram Biteye, Mame Thierno Bakhoum, Mamadou Ciss

**Affiliations:** 1 Laboratoire National de l’Elevage et de Recherches Vétérinaires, Institut Sénégalais de Recherches Agricoles, Dakar BP 2057, Senegal; mtseck@hotmail.fr (M.T.S.); bassenerose@yahoo.fr (M.D.B.); biteye88@yahoo.fr (B.B.); thierno.bakhoum@gmail.com (M.T.B.); ciss.mamadou@gmail.com (M.C.); 2 Département de Biologie Animale, Faculté des Sciences et Techniques, Université Cheikh Anta Diop, Dakar BP 5005, Senegal

**Keywords:** Sterile Insect Technique, marking method, fluorescent powder, mosquito handling

## Abstract

**Simple Summary:**

Marking techniques are generally used to differentiate colony mosquitoes from wild ones in the implementation of vector control programs using the Sterile Insect Technique. Different mosquito marking techniques have been developed in the past years but need improvement due to the extensive handling that can affect the quality of mosquitoes. We present here a self-marking technique that can reduce the damage associated with mosquito handling in mass rearing. The marking technique consists of adding fluorescent powder (DayGlo: A-17-N Saturn yellow) directly to the surface water of the receptacle containing *Aedes aegypti* male pupae. The marking efficacy, powder persistence and mosquito survival were assessed for male mosquito. We observed a high marking rate that increased with increasing amounts of fluorescent powder. Fluorescent powder lasted long on the mosquito body and did not induce a negative effect on their survival. This self-marking method reduces human intervention and mosquito handling, improving the quality of marked mosquitoes.

**Abstract:**

In the implementation of mosquito control strategy programs using Sterile Insect Technique and other rear and release strategies, knowledge on the dispersion, competitiveness and survival of mosquitos is considered essential. To assess these parameters, marking techniques are generally used to differentiate colony mosquitoes from wild ones. Most of the existing mosquito marking methods require numerous manipulations that can impact their quality. In this study, we have developed a self-marking technique that can reduce the damage associated with mosquito handling. The marking technique consisted of adding fluorescent powder (DayGlo: A-17-N Saturn yellow) directly to the surface water of the receptacle containing *Aedes aegypti* male pupae. Different quantities of powder were used, and marking efficacy, powder persistence and mosquito survival were assessed. The results show a mean marking rate of 98 ± 1.61%, and the probability of marking increased significantly (*p* < 0.001) with increasing concentrations of fluorescent powder. Fluorescent powder persisted up to 20 days and did not induce a negative effect on mosquito survival (χ^2^ = 5.3, df = 7, *p* = 0.63). In addition, powder transfer did not occur between marked and unmarked populations. This marking method significantly reduces human intervention and mosquito handling during the marking process, improving the quality of marked mosquitoes used to assess SIT programs.

## 1. Introduction

In the face of global and environmental change, many tropical and subtropical countries are confronted with major public health crises linked to the emergence/re-emergence of mosquito-borne diseases. Mainly transmitted by *Aedes* mosquitoes, Dengue virus (DENV) is the most extensive mosquito-borne pathogen affecting humans [1]. In late 2018, Senegal experienced its largest dengue virus outbreak to date, covering several regions [2]. As effective medical treatments and vaccines do not exist for DENV and most mosquito-borne diseases, vector control is essential and remains the main form of outbreak prevention [3].

Traditionally, vector control programs were focused on eliminating mosquitoes using various insecticides [4,5]. However, insecticide resistance was noted in many mosquito vectors, including *Aedes aegypti* [6,7,8]. This species has been found to be resistant to the majority of insecticide classes [9]. These findings and the problems of environmental pollution linked to the use of insecticides encouraged the development of alternative eco-friendly methods such as the Sterile Insect Technique (SIT) [10,11,12,13], the Incompatible Insect Technique (IIT) [14,15,16] and gene drive systems [17]. Thus, estimating the bio-ecological parameters of *Ae. aegypti* is crucial to implementing vector control measures based on these new control strategies. Mosquito dispersal, competitiveness, and survival are key parameters to be determined when implementing these control methods in the field. These parameters are most often evaluated using mark–release–recapture (MRR) techniques [18]. In the past, marking was used to study the vector behaviour to estimate its role in pathogen transmission [18]. Currently, marking is often applied to male mosquitoes in new control strategies [19,20]. Most of the time, the use of these techniques is required prior to release [21]. The effectiveness of the MRR technique depends on the sensitivity of the capture methods and reliability of the marking method. Several mosquito marking techniques exist, including tags, mutilation, dye, dust, isotopes, genetic, radioactive-isotope, and protein marking [22]. The ideal marker should persist without changing the biology of the vector and be environmentally friendly, cost-effective, and easily applied [21]. Usually, adult mosquitoes are marked by blowing small amounts of fluorescent dust pigments or powders on the external surface of the body or by creating a dust cloud in a closed container [23,24]. With certain marking methods, mosquitoes are subjected to immobilisation treatment at low temperature [19], which can lead to high mortality or other negative impacts on their biology [25,26]. Additionally, the incorrect application of dust and powder can increase mortality, decrease mobility, and affect the sensory organs, giving biased results in MRR studies [24,25]. Poor persistence and the potential transference of the dust or power to unmarked individuals imparts additional confounding effects.

Marking techniques that do not require mosquito handling help to reduce the biological costs of the traditional marking method. Such marking methods have been developed for different insects, including mosquitoes, in both laboratory and field settings [27,28,29]. Non-handling methods for mosquitoes have been developed for radioisotopes [30,31,32], food colours [33,34,35], and fluorescent powder [36]. All these marking techniques require the use of specialized equipment to mark and sort positive and negatively marked mosquitoes [36]. Such equipment can be costly, difficult to handle, and/or harmful for the environment and the manipulator.

Within the framework of control strategies using an SIT component reliant on repeated releases of large numbers of sterile males, it is necessary to develop marking methods that do not significantly reduce the quality of marked insects. Currently, new technologies and knowledge are available to support these efforts as part of area-wide integrated pest management (AW-IPM) programs [37,38]. Herein, we detail the development of a self-marking technique developed for *Ae. aegypti* mosquitoes to be used in the assessment of SIT programs and other vector control initiatives. The marking method described is inspired by the self-marking method used in the eradication program of *Glossina palpalis gambiensis* in the Niayes area of Senegal, where pupae were buried in a mixture of sand and fluorescent powder in order to mark teneral insects at their emergence [39,40]. The method simulates the natural environment of pupae that are buried in the ground at varying depths [41]. Comparatively, mosquito pupal development occurs in an aquatic medium. Therefore, in this study, we applied the principle of the self-marking technique developed for *G. palpalis gambiensis* to develop a self-marking method for *Ae. aegypti* and potentially other medically and economically important mosquito species.

## 2. Materials and Methods

### 2.1. Mosquito Rearing Conditions

All experiments were carried out at the insectarium of the Laboratoire National de l’Elevage et de Recherches Vétérinaires (LNERV) at the Institut Sénégalais de Recherches Agricoles (ISRA) (14.7224794; −17.4333597). The temperature and relative humidity conditions in the rearing rooms were 25 ± 1 °C and 75 ± 5%, respectively, with a light/dark photoperiod of 12:12 h. A data logger (thermo-hygrometer HOBO; Onset, Pocasset, MA, USA) was hung in the rooms to continuously monitor these parameters. The *Ae. aegypti* strain used in the study has been maintained under LNERV-ISRA insectarium conditions since 2015. The larvae were fed with 35 g/75 mL Mikrovit^®^ powder (TROPICAL Tadeusz Ogrodnik. ul., Chorzów, Poland). The pupae were collected each morning, sexed using a Fay-Morlan glass plate sex separator [42,43], placed in plastic containers with water (300 mL), and then transferred to adult cages that were locally manufactured. The cages were metal structures measuring 30 cm × 30 cm × 30 cm covered by a mosquito net with an opening sleeve for mosquito handling. Adults (males and females) were continuously fed with 10% sucrose solution. Once a week, females were fed with sheep’s blood using a membrane feeding system (Hemotek Ltd., Blackburn, UK).

### 2.2. Mosquito Self-Marking

The marking method consisted of spreading a selected amount of fluorescent powder (DayGlo Color Corp, Cleveland, OH, USA) on the surface of the water of the plastic cup containing 1000 pupae in ca. 500 mL of water. The cups were then placed in emergence cages (30 cm × 30 cm × 30 cm). Different fluorescent powder quantities (100 mg; 200 mg; 300 mg; 400 mg; 500 mg; 1000 mg and 1500 mg) were tested. The total area of water covered by the fluorescent powder was estimated at 90.25 square centimetres (9.5 cm × 9.5 cm). Subsequently, adult mosquitoes emerging from pupae were marked by fluorescent powder. Each emergence cage was labelled with the following information: date, quantity of powder, and the replicate number. An ultra-violet (UV) camera “DinoCapture” version 2.0 (AnMo Electronic Corporation, Hsinchu City, Taiwan) was used to check the presence of fluorescent powder on the whole body of each individual mosquito upon emergence. The main steps of the marking procedure are summarised as follows: (1) separation of male and female mosquito pupae, (2) spreading fluorescent powder on the surface of the water of the plastic box containing only male mosquito pupae, (3) emergence of mosquito inside the emerging cage, and (4) observation of the presence of fluorescent powder on the body of the mosquito.

#### 2.2.1. Evaluation of the Marking Effectiveness

The emergence rate was calculated for each treatment to assess the impact of the powder to the developing pupae. Then, in order to determine the effectiveness of the marking for each treatment, 100 individuals were picked at random 3 days after emergence, killed by freezing at −20 °C, and then scored for the presence of fluorescent powder using the UV camera. Scoring of marking effectiveness was noted as “1” if powder was present at any part of the mosquito body (i.e., head, thorax, abdomen, legs or wings) and “0” if the powder was absent from any part of the mosquito body. Three replicates were performed for each treatment from three different containers.

#### 2.2.2. Assessment of Fluorescent Powder Transfer to Female

Fluorescent powder transfer from marked males to unmarked females during mating was tested. Using a mouth aspirator (John W. Hock Company, Gainesville, FL, USA), 25 3 day old marked males with the highest fluorescent powder dose (1500 mg) and 25 unmarked virgin females of *Ae. aegypti* were placed in the same cage (30 cm × 30 cm × 30 cm) for 3 days. They were fed with 10% sucrose solution. All females were killed by freezing at day 4 at −20 °C and observed using the UV camera in order to detect fluorescent powder. Three replicates were performed.

#### 2.2.3. Assessment of Males’ Survival and Fluorescent Powder Persistence

To assess the effect of the marking technique on male survival, daily mortality was recorded in each dose (100 mg; 200 mg; 300 mg; 400 mg 500 mg; 1000 mg and 1500 mg). Survival was determined by transferring 30 marked male mosquitoes into individually labelled plastic cups (570 mL). The cups were covered with a mosquito net, and males were provided with 10% sucrose solution ad libitum. Dead mosquitoes were recorded and removed from the plastic cup every morning for a period of 20 days. To evaluate the persistence of the fluorescent powder on male mosquitoes, dead mosquitoes collected during the survival experiment were scored for the presence of fluorescent powder upon collection. At the end of 20 days, surviving mosquitoes were frozen and observed to detect the presence of fluorescent powder on their bodies. Three replicates were performed for both experiments.

### 2.3. Statistical Analyses

The effectiveness of the marking method was calculated as the number of mosquitoes observed with fluorescent powder by the total number of mosquitoes scored (n = 100). The survival of marked mosquitoes from different treatments was analysed using Kaplan–Meier survival curves. The log-rank test (Mantel–Cox) [44,45,46] was used to compare the survival rate between the different treatments in each experiment. The daily mortality rate (DMR) was calculated as the percentage of dead mosquitoes observed every 24 h in each treatment [47]. Binomial linear mixed effect models were used to analyse the impact of the various doses of fluorescent powder used on daily survival rates. Doses were then used as fixed effects, and the replication and date of experiment as random effects. The quantity of 00 mg was set as a reference level (control) in the model, and other treatments were compared to this value. The odds ratios (OR) with 95% confidence intervals (CI) were calculated to assess the probability that a mosquito was marked according to the amount of powder (100 mg; 200 mg; 300 mg; 400 mg 500 mg; 1000 mg and 1500 mg for 1000 pupae) applied to surface water. For any value of OR > 1, the probability of observing the fluorescence (event) was OR-value times greater than those of a non-marking event; for OR = 1, the probability of observing the event was equal to that of a non-marking event; and for OR < 1, the probability of observing a marked mosquito was (1 − OR) × 100 times smaller than those of non-event at OR = 1. All statistical analyses were carried out with R software [48], and log-rank tests were performed using survival and survminer R packages [49].

## 3. Results

### 3.1. Effectiveness of the Self-Marking Method and Dust Contact-Transfer

**Figure 1:**
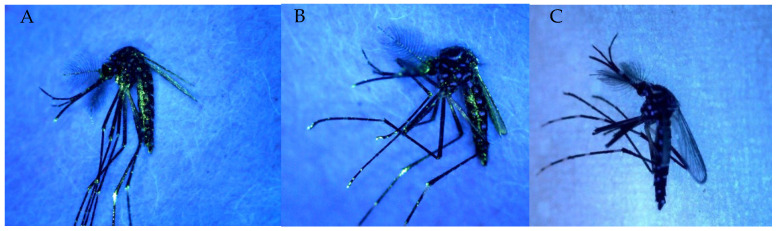
Pictures of marked (500 mg powder) 1 day old (**A**), 20 days old (**B**) and unmarked 3 days old (**C**) *Aedes aegypti* with the self-marking method using UV camera. The marking appears in green florescent spots on the mosquito’s body.

We found that there was no significant difference in emergence rates between the controls and all the treatment doses. On average, the adult emergence rate was 98.45% (SE = 0.58) (Table 1). Overall, the mean percentage of marked mosquitoes across all treatments was 98% (SE = 1.61). Fluorescent powder was present on the whole body of the mosquito and easily readable under the UV camera (Figure 1) for all treatments. The percentage of marked mosquitoes ranged from 96.33% (CI = 0.94) to 99.33% (SE = 0.94) across all treatments. Despite high rates of marking, the probability of successful marking was found to vary significantly depending on the quantity of powder used (*p* < 2 × 10^−16^). Compared to the lowest quantity (100 mg), the probabilities of the mosquitoes being marked were 1 to 3 times greater when using 200 mg (CI = 0.458–2.687), 300 mg (CI = 0.618–4.383), 400 mg (CI = 0.7–5.475), and 500 mg (CI = 0.951–10.255), and five times greater when using 1000 mg (CI = 1.506–36.858) and 1500 mg [CI = 1.506–36.858].

We did not observe dust transfer by contact from the marked males to the unmarked females when using even the highest quantity of dust.

### 3.2. Survival of Marked Males and Fluorescent Powder Persistence

**Figure 2:**
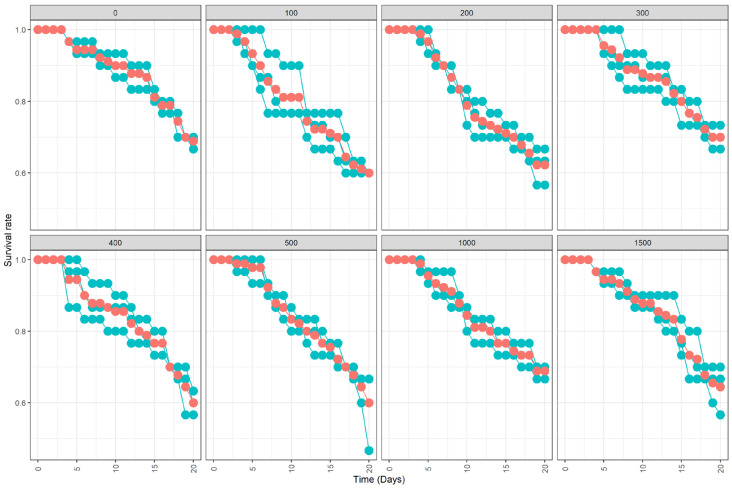
Mean (±standard error, SE) % survival of adult male *Aedes aegypti* 20 days following exposure to green florescent powder at 0, 100, 200, 300, 400, 500, 1000, and 1500 mg. Mean values are represented by red circles and outlined. Each series of dark olive-green dots represents an individual replicate (30 adult males).

No negative impact on the survival of mosquitoes regardless of the quantity of powder used was observed (Table 2; Figure 2, χ^2^ = 5.3, df = 7, *p* = 0.63). However, the mean daily mortality rate differed significantly between the control and all treatments (χ^2^ = 11.1, df = 7, *p* = 0.135).

Concerning the persistence of the florescent powder on mosquitoes, observation at time of death showed that the powder persisted in 100% of male mosquitoes (n = 630) throughout the duration of the experiment (20 days; Figure 1).

## 4. Discussion

In this paper, we present a new mosquito marking method that can eliminate or reduce handling by relying on the application of fluorescent powder directly to the pupal rearing water. To our knowledge, this is the first time that such an approach has been used for the marking of male mosquitoes. Our results show a high marking efficacy of 98% across all treatments, with equally high persistence of the mark without any significant negative impact on male survival in laboratory conditions. This method negates the need for extra manipulation of male mosquitoes, such as chilling before marking adults [19]. Reduced handling maintains the physical integrity of the mosquitoes, which in turn minimizes impacts on their quality [25]. Our method used much more fluorescent powder per individual than reported previously [19], but its application to surface water did not induce additional fitness costs and provided discreet coloration that was easily observable under a UV light camera. This self-marking method can be suitable for assessing SIT programs and other mosquito interventions. Similar approaches have been used successfully in *Glossina palpalis gambiensis* [40] and *Dendroctonus ponderosae Hopk*. (Coleoptera: Scolytidae) [50].

Our results show that the rate of coloured mosquitoes increased but did not differ significantly with the increase of the quantity of powder. Comparatively, McMullen et al., (1988) found almost similar marking rates using a similar method with *Dendroctonus ponderosae* [50]. These authors observed that 99.1% of the beetles that emerged from the treated bolts were marked [50]. Moreover, Niebylski et al. [29] found 100% marked *Culex* using a self-marking device. This device was equipped with cheesecloth partitions impregnated with the pigment. In contrast, Saddler and al. [51] found that 86% of *Anopheles arabiensis* emerging from their self-marking device were marked. Authors using self-marking techniques to mark insects have reported lower rates than ours [52,53]. However, the effectiveness of our self-marking technique should be tested for other mosquito genera and species for standardisation, as shown by Culbert et al. [31].

Importantly, our self-marking method showed no transference of dust from the marked mosquitoes to unmarked female mosquitoes after three days of contact in the emergent cages. The lack of transference may be explained by the fact that the quantity of powder deposited on the mosquito body in our method is less than that when powder is actively applied directly to adult mosquitoes. Although, to confirm, the lack of transference of the mark could allow a reduction of the risk of false positive mosquitoes in case of MRR assessment of SIT programs. Culbert et al. [31] found with their marking method that there is a chance of dust transfer to the unmarked mosquito, which was noticeable only under a stereomicroscope [19].

Lastly, we found that there was no significant difference in male survival between control and treatment groups. Our observations agree with those of Niebylski and Meek on *Culex quinquefasciatus* [29] and those of Saddler et al. on the malaria vector *Anopheles gambiae* sensu lato (s.l.) [51]. In contrast, the bag and bulb duster marking method has been shown to significantly affect the survival of males and females of *Ae. aegypti* [25]. Similarly, Verhulst et al. [30] showed that applying fluorescent dyes or powders directly to mosquitoes that were at least 5 days old significantly reduced their survival probabilities [24]. Similar results were observed by Culbert et al. [19]. This could be explained by the fact that in certain marking methods (i.e., bag and bulb duster), excess powder is directly applied to adult mosquitoes, which can interfere with the sensoria’s organs and induce immediate mortalities and/or affect their longevity [31,34,37]. Our self-marking method needs less time and reduces the need to handle mosquitoes, resulting in high-quality marked mosquitoes at the time of release.

## 5. Conclusions

This study has allowed us to set up a new, innovative, self-marking method that is easily applicable in SIT programs. The self-marking method significantly reduces mosquito handling during the process, which can impact positively the quality of marking. The results indicate that no significant difference was found regarding the survival of marked and unmarked mosquitoes, and powder transfer did not occur between the two populations. The 3 week follow up of marked mosquitoes showed that they remained coloured and easily detectable under a UV-light camera. However, further studies are needed to assess the method in field conditions and on other mosquito species for its standardisation.

## Figures and Tables

**Table 1:** Emergence rate and efficacy of the self-marking method as a function of the quantity of fluorescent powder applied to surface water.

Quantity of Powder (mg)	Control	1500	1000	500	400	300	200	100	Mean
Emergence rate + SE	98.13 ± 0.55	98.83 ± 0.35	98.86 ± 0.25	98.60 ± 0.61	98.53 ± 0.76	98.66 ± 0.21	98.17 ± 0.61	98.07 ± 1.04	98.45 ± 0.58
Rate of males marked + SE	00 ± 0.00	99.33 ± 0.94	99.33 ± 0.47	98.67 ± 1.24	98 ± 1.41	97.67 ± 1.24	96.67 ± 1.24	96.33 ± 0.94	98 ± 1.61
Daily mortality rate	0.0172 ± 0.0011	0.0213 ± 0.0044	0.0170 ± 0.0013	0.0238 ± 0.0072	0.0234 ± 0.0018	0.0165 ± 0.0018	0.0219 ± 0.0031	0.0234 ± 0.0002	0.0218 ± 0.28

SE = standard error.

**Table 2:** Mixed-effect binomial model of male *Aedes aegypti* survival in function of fluorescent powder quantity.

Fluorescent Powder Quantity (mg)	Estimate	Std. Error	z Value	Pr (>|z|)
Control	2.037	0.394	5.165	2.399 × 10^−7^
100	−0.594	0.508	−1.168	0.242
200	−0.524	0.513	−1.021	0.306
300	−0.109	0.546	−0.200	0.841
400	−0.362	0.524	−0.689	0.490
500	−0.354	0.525	−0.673	0.501
1000	−0.254	0.533	−0.476	0.634
1500	−0.211	0.537	−0.392	0.695

Std = standard deviation; mg = milligram.

## Data Availability

The datasets presented in this study are available on request from the corresponding author.
